# Amphetamine-induced reverse transport of dopamine does not require cytosolic Ca^2+^

**DOI:** 10.1016/j.jbc.2023.105063

**Published:** 2023-07-18

**Authors:** Jonatan Fullerton Støier, Ainoa Konomi-Pilkati, Mia Apuschkin, Freja Herborg, Ulrik Gether

**Affiliations:** Molecular Neuropharmacology and Genetics Laboratory, Department of Neuroscience, Faculty of Health and Medical Sciences, Panum Institute - Maersk Tower 7.5, University of Copenhagen, Copenhagen, Denmark

**Keywords:** amphetamine, dopamine transporter, calcium signaling, genetically encoded dopamine sensor, biosensors, live fluorescent imaging

## Abstract

Amphetamines (AMPHs) are substrates of the dopamine transporter (DAT) and reverse the direction of dopamine (DA) transport. This has been suggested to depend on activation of Ca^2+^-dependent pathways, but the mechanism underlying reverse transport *via* endogenously expressed DAT is still unclear. Here, to enable concurrent visualization by live imaging of extracellular DA dynamics and cytosolic Ca^2+^ levels, we employ the fluorescent Ca^2+^ sensor jRGECO1a expressed in cultured dopaminergic neurons together with the fluorescent DA sensor GRAB_DA1H_ expressed in cocultured “sniffer” cells. In the presence of the Na^+^-channel blocker tetrodotoxin to prevent exocytotic DA release, AMPH induced in the cultured neurons a profound dose-dependent efflux of DA that was blocked both by inhibition of DAT with cocaine and by inhibition of the vesicular monoamine transporter-2 with Ro-4-1284 or reserpine. However, the AMPH-induced DA efflux was not accompanied by an increase in cytosolic Ca^2+^ and was unaffected by blockade of voltage-gated calcium channels or chelation of cytosolic Ca^2+^. The independence of cytosolic Ca^2+^ was further supported by activation of N-methyl-D-aspartate-type ionotropic glutamate receptors leading to a marked increase in cytosolic Ca^2+^ without affecting AMPH-induced DA efflux. Curiously, AMPH elicited spontaneous Ca^2+^ spikes upon blockade of the D2 receptor, suggesting that AMPH can regulate intracellular Ca^2+^ in an autoreceptor-dependent manner regardless of the apparent independence of Ca^2+^ for AMPH-induced efflux. We conclude that AMPH-induced DA efflux in dopaminergic neurons does not require cytosolic Ca^2+^ but is strictly dependent on the concerted action of AMPH on both vesicular monoamine transporter-2 and DAT.

Amphetamine (AMPH) is both a widely abused drug and a drug commonly prescribed to treat attention-deficit hyperactivity disorder (1, 2). The psychostimulatory properties of AMPH are attributed to its interaction with the dopamine transporter (DAT) that regulates dopamine (DA) signaling by transporting DA back into the presynaptic neuron (3). AMPH is a substrate of DAT and causes nonexocytotic efflux of DA by reversing the direction of transport *via* DAT through a yet not fully understood mechanism (4, 5, 6, 7). AMPH is also a substrate of the vesicular monoamine transporter 2 (VMAT2) that sequesters DA into synaptic vesicles (8). It has been suggested that the exchange of AMPH for DA through the VMAT2 may deliver DA for efflux through DAT (7, 9, 10, 11). Notably, VMAT2 operates as a proton antiporter and relies on the low luminal pH to transport DA into the synaptic vesicles for a high luminal concentration (∼0.5 M) (12, 13). Transport of AMPH into the vesicles, however, is likely to disrupt the proton gradient, as AMPH may alkalize the vesicle lumen and cause accumulation of DA in the cytosol, which might facilitate reverse transport of DA out of the cell *via* DAT (11, 12, 14).

The mechanism underlying AMPH-induced efflux *via* DAT has been suggested to involve several intracellular signaling pathways. Specifically, intracellular Ca^2+^ has been suggested to play a vital role. In heterologous cells overexpressing DAT, AMPH promoted an increase in cytosolic Ca^2+^ and AMPH-induced efflux was impaired by chelating cytosolic Ca^2+^ (15). A requirement for intracellular Ca^2+^ was also observed for AMPH-induced efflux via the norepinephrine transporter in cultured PC12 cells (16). In cultured midbrain neurons, methamphetamine was found to increase cytosolic Ca^2+^, but the implications for AMPH-induced efflux were not settled (17). Of further interest, both PKC, including the Ca^2+^-dependent ß-subtype (16, 18, 19, 20, 21, 22, 23, 24), and Ca^2+^/calmodulin-dependent protein kinase IIα (CAMKIIα) have been implicated in the process (25, 26, 27, 28, 29). The data have suggested that phosphorylation of N-terminal serines changes the transporter to a “willing state” for efflux (21). This phosphorylation can be mediated by CaMKIIα and facilitated by an interaction of the kinase with the DAT C-terminus (25). AMPH-induced efflux might also be regulated by the interaction of DAT with syntaxin1A (30), G protein βγ subunits and the neuronal GTPase, Rit2 (31, 32, 33), as well as by lipid messengers (*e.g*., phosphatidylinositol 4,5-bisphosphate) (34). Taken together, multiple mechanisms have been implicated in AMPH-induced efflux; however, since many studies have been done in heterologous cells, the picture is still blurry as to what mechanisms represent the key drivers of efflux in dopaminergic (DArgic) neurons (35).

To improve our insights into AMPH-induced efflux with a focus on the unsettled role of Ca^2+^, we establish here a novel approach enabling simultaneous assessment of DA efflux and intracellular signaling pathways by live fluorescent imaging of cultured DA midbrain neurons. We combine targeted expression of genetically encoded fluorescent biosensors in DArgic neurons with seeding of the culture with “sniffer” HEK293 cells (36) expressing the fluorescent DA sensor GRAB_DA1H_ (37) to record extracellular DA dynamics with high sensitivity and temporal resolution. In summary, our data provide strong evidence that AMPH-induced DA efflux *via* DAT in DArgic neurons does not require Ca^2+^ but primarily relies on the concerted action of AMPH on VMAT2 and DAT. AMPH promoted nonetheless spontaneous Ca^2+^ spikes upon blockade of the D2 receptor, suggesting that AMPH can regulate intracellular Ca^2+^ in an autoreceptor-dependent manner despite no apparent effect of Ca^2+^ on efflux. In addition, to shed new light on the role of Ca^2+^ in AMPH-induced DA efflux, our results provide an important methodological framework for further future studies of the complex actions of AMPHs on DA neurons.

## Results

### A novel experimental approach to study DA release concomitantly with cytosolic Ca^2+^ in cultured DArgic neurons

**Figure 1:**
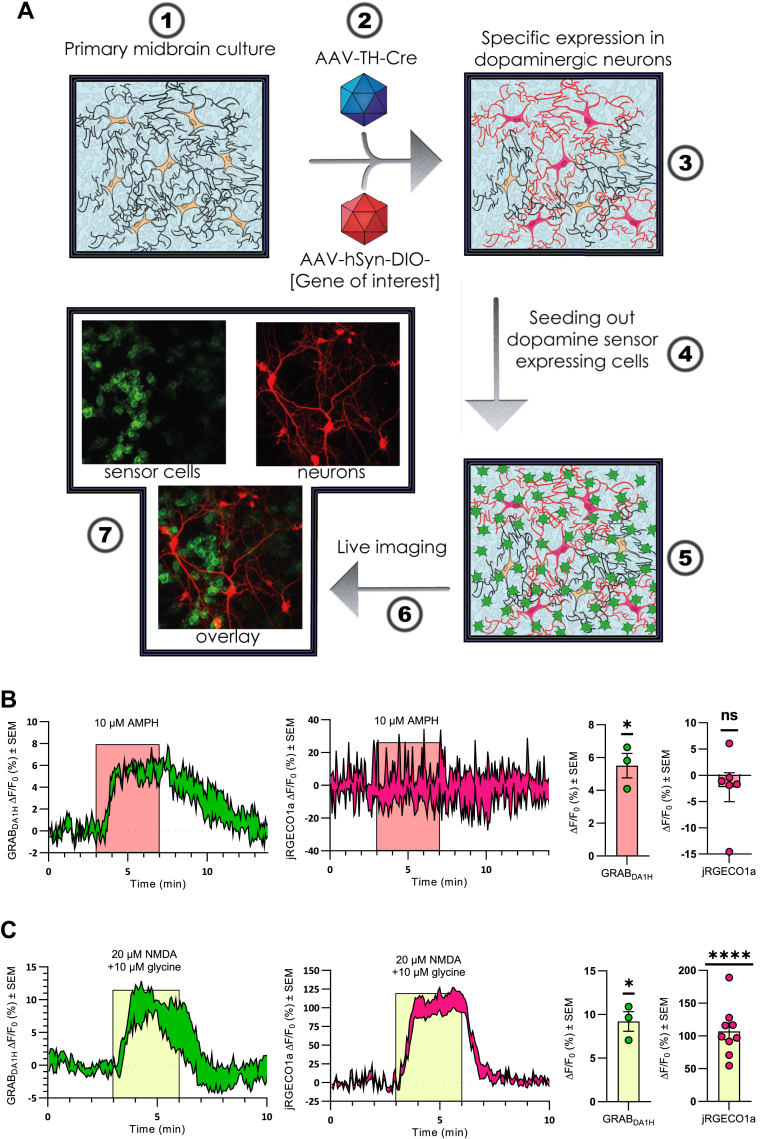
**A novelexperimental approach to study DA release combined with selective expression of the calcium sensor jRGECO1a in cultured DArgic neurons**. The experiments suggest that AMPH-induced DA efflux occurs in the absence of change in intracellular Ca^2+^. *A*, graphical illustration of the approach with the numbering of each step as follows: (1), midbrain neurons from postnatal rat pups are seeded on a glia monolayer (2), a dual viral approach is exploited where one AAV mediates tyrosine hydroxylase promoter-driven Cre expression and a second AAV mediates the Cre-dependent expression of a gene of interest (3), this allows targeted expression of the gene in DArgic neurons (4), one to two days before imaging, HEK293 cells stably expressing the DA sensor GRAB_DA1H_ are seeded on top of the culture (5), the DA sensor expressing cells have attached to the culture the day after being seeded and stay well attached during live imaging with constant flow (6), the DA sensor in the HEK293 cells allows for a good signal-to-noise ratio and makes it possible to easily separate the signal of the DA sensor from the sensor expressed in the neurons. *B*, change in DA signal from GRAB_DA1H_ (*left*) and Ca^2+^ signal from jRGECO1a (*middle*) upon application of 10 μM AMPH as indicated. Data shown are an average trace of three independent experiments ± SEM. The bar graphs (*right*) represent the average change in fluorescence during the last minute of AMPH from baseline. Data show that AMPH causes a change in the fluorescent DA signal, but not the Ca^2+^ signal. Data are mean of ΔF/F_0_ in % ± SEM, n = 3 for GRAB_DA1H_ and n = 6 for jRGECO1a. *C*, change in DA signal from GRAB_DA1H_ (*left*) and Ca^2+^ signal from jRGECO1a (*middle*) upon applying 20 μM NMDA and 10 μM glycine as indicated. Data shown are an average trace of three independent experiments ± SEM. The bar graphs (*right*) represent the average change in fluorescence during the last minute of NMDA treatment from baseline. Data are mean of ΔF/F_0_ in % ± SEM, n = 3 for GRAB_DA1H_ and n = 9 for jRGECO1a. ns, *p* > 0.05; ∗*p* ≤ 0.05; ∗∗∗∗*p* < 0.0001; one-sample *t* test. AAV, adeno-associated virus; AMPH, amphetamines; DA, dopamine; NMDA, N-methyl-D-aspartate.

To measure AMPH-induced DA efflux in cultured DArgic neurons, we used T-REx-293 cells stably expressing the genetically encoded single fluorophore- and D2 receptor-based DA sensor GRAB_DA1H_ (36). To simultaneously monitor DA efflux and Ca^2+^ responses with negligible spectral and spatial overlap, we combined these GRAB_DA1H_ -expressing “sniffer” cells with a dual virus approach for targeted expression of the red fluorescent Ca^2+^ sensor jRGECO1a in DArgic neurons (Fig. 1*A*). Primary midbrain cultures were transduced with two adeno-associated viruses (AAVs) of which one encoded Cre recombinase under the control of a truncated tyrosine hydroxylase promotor (AAV-pTH-iCre-WPREpA) and the other Cre-dependent expression of jRGECO1a (AAV-CAG-Flex-NES-jRGECO1a-WPREpA) (38) (for specificity of this approach, Fig. S1).

Before imaging, the sniffer cells were seeded on top of the midbrain cultures. As expected, application of AMPH (10 μM) caused an increase in fluorescence from GRAB_DA1H_ (ΔF/F_0_ (%) ± SEM = 5.5 ± 0.75; *p* = 0.018, n = 3, one-sample *t* test) indicating a surge in extracellular DA (Fig. 1*B*). The increase in DA caused by AMPH was reversible as the fluorescence returned to baseline following washout (Fig. 1*B*). The increase was not caused by AMPH interacting with the GRAB_DA1H_ sensor itself because no increase in fluorescence was observed by directly exposing sniffer cells to AMPH (Fig. S2). In the coculture experiment, no effect of AMPH was seen on cytosolic Ca^2+^-levels, that is, the change in jRGECO1a fluorescence was not different from baseline (ΔF/F_0_ (%) ± SEM = −2.3 ± 2.7; *p* = 0.45, n = 6, one-sample *t* test) (Fig. 1*B*).

To illustrate Ca^2+^-dependent vesicular DA release promoted by excitatory input, we stimulated the neurons with 20 μM N-methyl-D-aspartate (NMDA) plus 10 μM glycine, resulting in an increase in GRAB_DA1H_ signal (ΔF/F_0_ (%) ± SEM = 9.2 ± 1.1; *p* = 0.015, n = 3, one-sample *t* test) (Fig. 1*C*). In contrast to what we observed for AMPH, this increase was accompanied by a large rise in intracellular Ca^2+^ that followed the same time course as the rise in extracellular DA levels (ΔF/F_0_ (%) ± SEM = 107 ± 13; *p*< 0.0001, n = 9, one-sample *t* test) (Fig. 1*C*).

### Dose-dependent AMPH-induced efflux is not accompanied by an increase in cytosolic Ca^2+^

**Figure 2:**
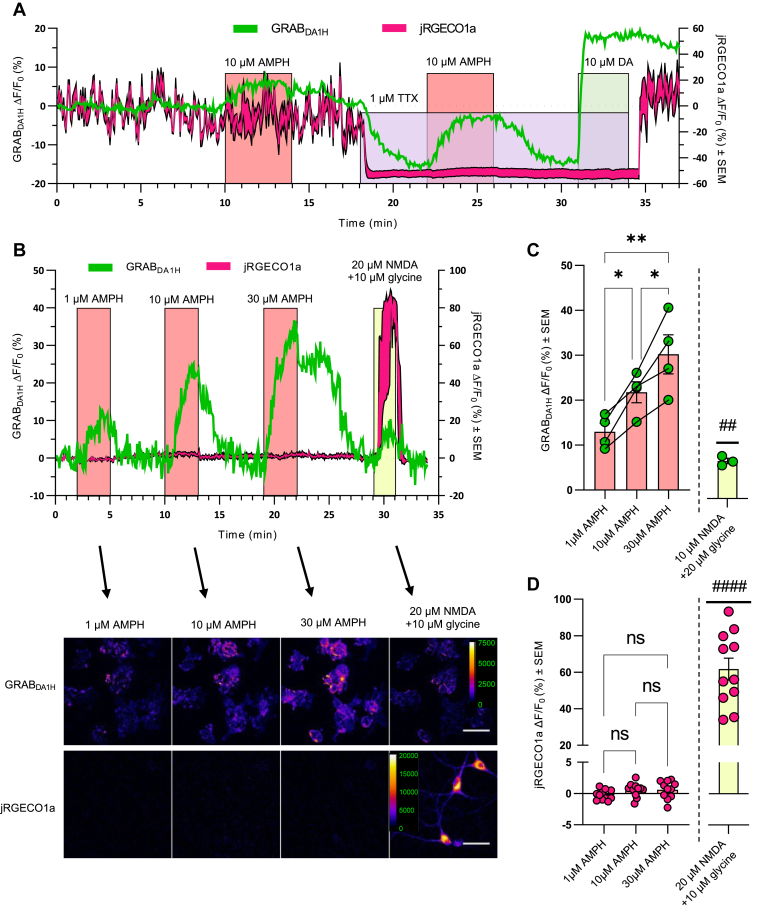
**Dose-dependent AMPH-induced efflux still occurs in the presence of the voltage-gated sodium-channel blocker TTX and is not accompanied by an increase in cytosolic Ca**^**2+**^**.** The absence of spontaneous firing of the neuron allows for the isolation of the DA signal solely from the contribution of AMPH. In the presence of TTX, the effect of AMPH on the DA signal from GRAB_DA1H_ is dose-dependent, while no change in intracellular the Ca^2+^ signal from jRGECO1a is detected. *A*, representative trace of three independent experiments illustrating the effect of TTX on extracellular DA and intracellular Ca^2+^. The jRGECO1a trace is the average signal ± SEM of 5 somas in the recording. First, upon application of AMPH in the absence of TTX, an increase is seen in the DA signal, while it is unclear if it causes any change in the Ca^2+^ signal due to spontaneous fluctuation. Then, in the presence of 1 μM TTX the Ca^2+^ signal drops and becomes a flat line, and the DA signal decreases. Application of AMPH in the presence of TTX causes no change in the Ca^2+^ signal. Still, it induces an increase in the DA signal with a clearer signal than the signal in the absence of TTX. Finally, 10 μM DA is added to saturate GRAB_DA1H_ and thereby visualize the dynamic range of the fluorescent DA signal. *B*, (*top*) representative trace of dose-response experiment in the presence of TTX with 1, 10, and 30 μM AMPH followed by 20 μM NMDA and 10 μM glycine. The jRGECO1a trace is the average signal ± SEM of 3 somas in the recording. (*Bottom*) Representative images of the change in DA and Ca^2+^ signal from baseline during the last minutes of each of the drug applications as indicated by the *arrows*. The scale bar represents 50 μm. *C*, the bar graph shows the average change in DA signal compared to vehicle. Data are mean of ΔF/F_0_ in % ± SEM, with n = 4 for 1, 10, and 30 μM AMPH responses and n = 3 for NMDA response. ∗*p* ≤ 0.05, ∗∗*p* ≤ 0.01, one-way ANOVA with Holm–Šídák multiple comparison test (F _(2, 6)_ = 18.6, *p* = 0.0027); ##*p* < 0.01, one-sample *t* test. *D*, the bar graph shows the average change in Ca^2+^ signal from baseline. While there was no change in Ca^2+^ signal for the doses of AMPH tested, there was a clear change upon applying 20 μM NMDA + 10 μM glycine. Data are mean of ΔF/F_0_ in % ± SEM, n = 11.; ns, nonsignificant (*p* > 0.05), one-way ANOVA with Holm–Šídák multiple comparison test (F(2, 20) = 2.071, *p* = 0.15); ####*p* < 0.0001, one-sample *t* test. AMPH, amphetamine; DA, dopamine; NMDA, N-methyl-D-aspartate; TTX, tetrodotoxin.

To minimize the interference of spontaneous neuronal activity, we applied tetrodotoxin (TTX) to block voltage-gated sodium channels and inhibit neuronal firing. Upon application of TTX, the basal levels of both DA and Ca^2+^ markedly decreased (Fig. 2*A*). Moreover, the rapid fluctuations in fluorescence from the two sensors were reduced, particularly for the Ca^2+^ sensor. These observations are consistent with reduced tonic firing and a concomitant reduction in the vesicular release of DA from the cultured neurons. Importantly, although TTX strongly inhibited neuronal activity, it was still possible to measure a substantial increase in DA level upon addition of AMPH with an improvement in signal-to-noise ratio (Fig. 2*A*). Therefore, we decided to include TTX in the artificial cerebrospinal fluid (aCSF) for all further experiments, enabling us to isolate the effect of AMPH on DA levels without background noise from spontaneous neuronal firing.

We next determined the dose-dependency of AMPH on DA efflux and intracellular Ca^2+^ (Fig. 2, *B*–*D*). Application of 1, 10, or 30 μM AMPH caused an increase in extracellular DA (ΔF/F_0_ (%) ± SEM, 1 μM: 13.0 ± 1.8; 10 μM: 21.8 ± 2.3; 30 μM: 30.2 ± 4.4) with a dose-response relationship between AMPH and DA efflux (one-way ANOVA, F _(2, 6)_ = 18.6, *p* = 0.0027) and with a difference between all three doses tested (Holm–Šídák post hoc test, 1 μM *versus* 10 μM: *p* = 0.041; 1 μM *versus* 30 μM: *p* = 0.0027; 10 μM *versus* 30 μM: *p* = 0.041). On the contrary, the same doses of AMPH had no effect on jRGECO1a fluorescence (1 μM: −0.24 ± 0.25, *p* = 0.36; 10 μM: 0.57 ± 0.33, *p* = 0.12; 30 μM: 0.60 ± 0.43, *p* = 0.19 (ΔF/F_0_ (%) ± SEM, n = 11, one-sample *t* test)). Importantly, post treatment with NMDA confirmed not only an increase in both DA (ΔF/F_0_ (%) ± SEM = 8.4 ± 0.61; *p* = 0.0052, n =3, one-sample *t* test), but also a large increase in jRGECO1a fluorescence (ΔF/F_0_ (%) ± SEM = 62 ± 6.0; *p* < 0.0001, n = 11, one-sample *t* test) (Fig. 2, *B*–*D*). In summary, AMPH causes a marked dose-dependent efflux of DA, which is not accompanied by an increase in intracellular Ca^2+^.

**Figure 3:**
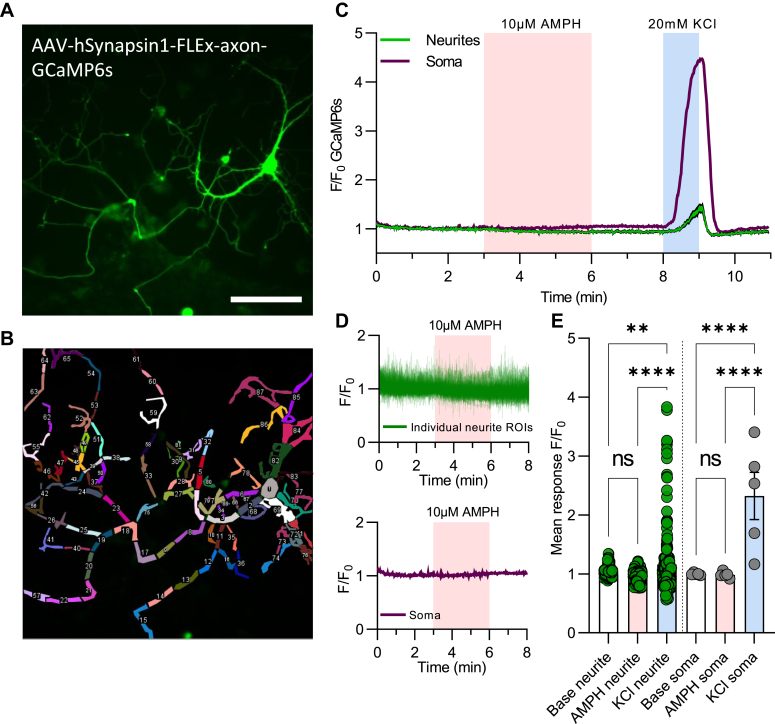
**AMPH does not induce an intracellular Ca**^**2+**^**increase in the neurites of DA neurons**. *A*, representative image of a DA neuron transduced with the axonally targeted Ca^2+^ sensor AAV-hSynapsin1-FLEx-axon-GCaMP6s expressed in Cre-expressing neurons, driven by the AAV-TH-Cre vector. The scale bar represents 100 μm. *B*, illustration of the individual ROIs selected for discrete Ca^2+^ measurements in the neurites compared to the soma. *C*, representative Ca^2+^ traces from the neurites and soma, plotted separately, of a neuron stimulated with 10 μM AMPH and 20 mM KCl. The neurites trace is the average signal ± SEM of the 86 neurite ROIs indicated as #2 to 87 in Figure 3*B*. *D*, enlarged traces from 3B during AMPH application. (*Top*) Plots of individual traces of the segmented neurites shown as ROI #2 to 87 in 3B. (*Bottom*) Plot of the soma trace shown as ROI #1 in *B*. *E*, comparison of mean responses to AMPH and KCl compared to baseline in individual neurite fractions (n = 199) and somas (n = 5). ∗*p* < 0.05, ∗∗∗*p* ≤ 0.001, ∗∗∗∗*p* ≤ 0.0001, one-way ANOVA with Holm-Šídák multiple comparison test (F_(5, 606)_ = 22.22, *p* < 0.0001). AAV, adeno-associated virus; AMPH, amphetamine; DA, dopamine; ROIs, regions of interest.

As the measurements with jRGECO1a primarily were done on the somatic compartment of the neurons, we decided to also use the axonally targeted sensor axon-GCaMP6s (39). Note that because of spectral overlap, these experiments could not be combined with simultaneous measurements of DA release using the GRAB_DA1H_ expressing sniffer cells. In DA neurons expressing the axon-GCaMP6s, there was a clear fluorescent signal, but we did not see a change in response to AMPH either in the neurites or the somatic compartment (Fig. 3), supporting the data obtained with jRGECO1a. A marked increase, however, was seen in response to a depolarizing concentration of KCl (Fig. 3).

### AMPH-induced DA efflux from DA neurons is blocked by cocaine and VMAT2 inhibition

**Figure 4:**
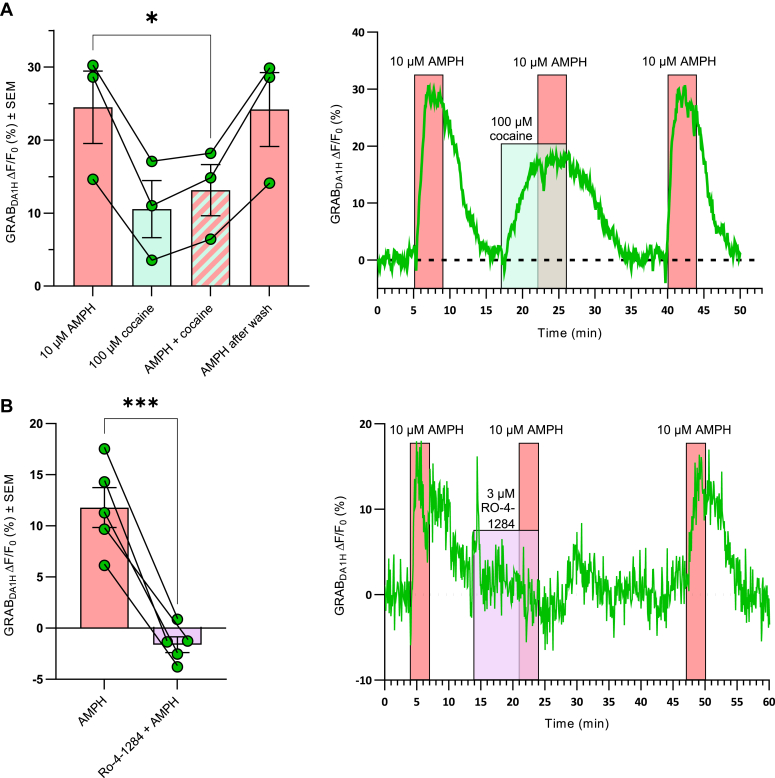
**The AMPH-induced DA efflux is both DAT and VMAT2 dependent in cultured midbrain DA neurons**. *A*, (*Left*) the bar graph shows the change in sniffer cell GRAB_DA1H_ signal from baseline upon adding, as indicated by the *colored areas*, 10 μM AMPH, 100 μM cocaine, 10 μM AMPH following preincubation with 100 μM cocaine, and lastly 10 μM AMPH following washout. Data are mean of ΔF/F_0_ in % ± SEM, n = 3. (*Right*) Representative trace from one of the recordings with colored backgrounds indicating drug treatments. *B*, (*Left*) The bar graph shows the change in sniffer cell GRAB_DA1H_ signal from baseline upon incubation with 10 μM AMPH alone or 10 μM AMPH together with the VMAT2 inhibitor Ro-4-1284. Data are mean of ΔF/F_0_ in % ± SEM, n = 3. (*Right*) Representative trace from one of the recordings with colored backgrounds indicating drug treatments. ∗*p* < 0.05, ∗∗∗*p* ≤ 0.001; paired *t* test. AMPH, amphetamine; DA, dopamine; DAT, dopamine transporter; VMAT2, vesicular monoamine transporter-2.

To test if the observed AMPH-mediated DA efflux in the presence of TTX occurred *via* DAT, 100 μM cocaine was applied prior and together with AMPH to block DAT (Fig. 4*A*). Cocaine alone increased extracellular DA (ΔF/F_0_ (%) ± SEM = 11 ± 3.9), which presumably reflected that cocaine prevents DAT-mediated reuptake of DA from the extracellular environment. Of significant importance, the effect of cocaine was markedly smaller than the effect of AMPH, and cocaine preincubation eliminated the AMPH-induced efflux of DA, directly supporting that the AMPH-induced efflux occurs *via* DAT (ΔF/F_0_ (%) ± SEM = 13 ± 3.5 from AMPH with cocaine and ΔF/F_0_ (%) ± SEM = 24 ± 4.9 without cocaine, *p* = 0.021, n = 3, paired *t* test) (Fig. 4*A*).

To assess the dependency on VMAT2 and vesicular DA for the action of AMPH, the VMAT2 blockers Ro-4-1284 and reserpine were investigated (Figs. 4*B* and S1). In presence of Ro-4-1284, the DA signal induced by AMPH was essentially abolished (ΔF/F_0_ (%) ± SEM = −1.6 ± 0.8 with and 12 ± 1.9 without Ro-4-1284; *p* = 0.0005, n = 5, paired *t* test). This effect was reversible, as the DA signal returned after washing with aCSF, suggesting that the diminished DA signal from blocking VMAT2 was not due to the emptying of DA from the synaptic vesicles (Fig. 4*B*). Importantly, reserpine was also able to inhibit AMPH-induced DA efflux (ΔF/F_0_ (%) ± SEM = 12 ± 9.8 with and 26 ± 5.6 without reserpine, *p* = 0.0023, n = 8, paired *t* test) (Fig. S3). Of note, the inhibition of VMAT2 by reserpine has previously been shown to be practically irreversible (40). In summary, our data suggest that AMPH-induced efflux is both DAT and VMAT2 dependent.

### AMPH increases spontaneous Ca^2+^ spikes upon inhibition of the D2 autoreceptor

**Figure 5:**
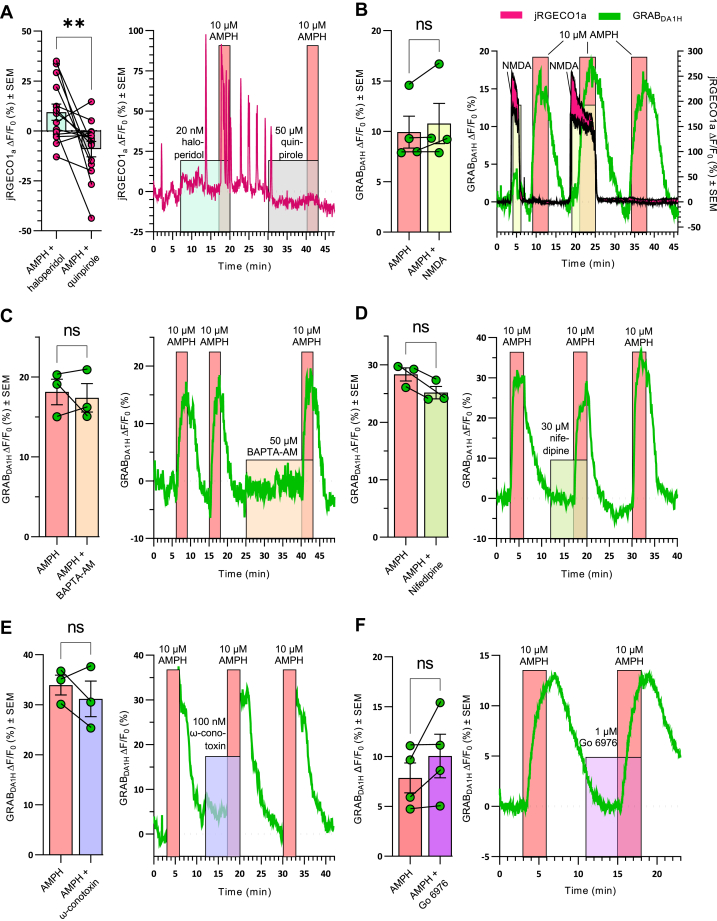
**AMPH increases spontaneous Ca**^**2+**^**spikes upon inhibition of the D2 autoreceptor**. Still, AMPH-induced DA efflux is not affected by increasing or chelating cytosolic Ca^2+^, blockade of voltage-gated Ca^2^+ channels or inhibition of PKC. *A*, 10 µM AMPH causes an increase in jRGECO1a Ca^2+^ spikes when coincubated with 20 nM of the D2 receptor antagonist haloperidol, while the opposite effect is seen in the presence of the D2 receptor agonist quinpirole (50 μM). (*Left*) Bar graph of the change in the Ca^2+^-dependent fluorescent signal of jRGECO1a from baseline during coapplication of AMPH with either haloperidol or quinpirole. Data are mean of ΔF/F_0_ in % ± SEM obtained from 14 neurons from four independent experiments. (*Right*) Representative trace of the effect of haloperidol and quinpirole on AMPH-induced increase in intracellular Ca^2+^ with colored backgrounds indicating drug treatments. *B*, preincubation with 20 μM NMDA and 10 μM glycine boost intracellular Ca^2+^ before application of AMPH did not affect AMPH-induced DA efflux. (*Left*) Bar graph showing the change in the DA-dependent fluorescent signal of GRAB_DA1H_ following application of AMPH with and without pre-incubation with NMDA. Data are mean of ΔF/F_0_ in % ± SEM, n = 3. (*Right*) Representative trace of the effect of NMDA on AMPH-induced DA signal from GRAB_DA1H_ and Ca^2+^ from jRGECO1a with colored backgrounds indicating drug treatments. The jRGECO1a trace is the average signal ± SEM of 3 somas in the recording. *C*, preincubating the neurons with 50 μM BAPTA-AM for 15 min to chelate intracellular Ca^2+^ did not affect DA efflux. (*Left*) The bar graph shows the change in the DA-dependent fluorescent signal of GRAB_DA1H_ following the application of AMPH with and without preincubation with BAPTA-AM. Data are mean of ΔF/F_0_ in % ± SEM, n = 3. (*Right*) representative trace of the effect of BAPTA-AM on AMPH-induced DA efflux. *D*, preincubation with 30 μM nifedipine for 5 min to block the L-type calcium channel did not affect DA efflux. (*Left*) The bar graph shows the change in DA fluorescent signal following the application of AMPH with and without preincubation with nifedipine. Data are mean of ΔF/F_0_ in % ± SEM, n = 3. (*Right*) Representative trace of the effect of nifedipine on AMPH-induced DA efflux. *E*, pre-incubation with 100 nM ω-conotoxin for 5 min to inhibit N-type calcium channels did not affect DA efflux. (*Left*) The bar graph shows the change in DA fluorescent signal following application of AMPH with and without preincubation with ω-conotoxin. Data are mean of ΔF/F_0_ in % ± SEM, n = 3. (*Right*) Representative trace of the effect of ω-conotoxin on AMPH-induced DA efflux. *F*, preincubation with the PKC inhibitor 1 μM Go 6976 for 3 min did not affect DA efflux. (*Left*) The bar graph shows the change in DA fluorescent signal following application of AMPH with and without preincubation with Go 6976. Data are mean of ΔF/F_0_ in % ± SEM, n = 4. (*Right*) Representative trace of Go 6976 on AMPH-induced DA efflux. ns, *p* > 0.05, ∗∗*p* ≤ 0.01; paired *t* test. AMPH, amphetamines; DA, dopamine; BAPTA, 1,2-bis(o-aminophenoxy)ethane-N,N,N′,N′-tetraacetic acid; NMDA, N-methyl-D-aspartate.

Previous data have suggested that AMPHs can cause an increase in cytosolic Ca^2+^ levels (15, 17, 41, 42). Given the absence of an effect of AMPH on cytosolic Ca^2+^ in our setup, we speculated whether the D2 autoreceptor could be constantly activated by DA release from the DArgic neurons. Stimulation of the D2 autoreceptor would expectably promote opening of G protein-coupled inwardly rectifying potassium channels (GIRKs) that, *via* hyperpolarization of the neurons, could prevent activation of voltage-gated Ca^2+^ channels (43, 44). The rise in DA observed in response to cocaine supported that DA release might occur even in the presence of TTX (Fig. 4*A*). We also sometimes observed spontaneous Ca^2+^ spikes in single individual neurons (Fig. 5*A*). Therefore, we examined the effect of the D2 receptor antagonist, haloperidol and the agonist quinpirole. Strikingly, in the presence of 20 nM haloperidol, we observed spontaneous Ca^2+^ spikes for individual neurons following AMPH treatment (Fig. 5*A*). Quantification of the AMPH-induced jRGECO1a response substantiated an increase in cytosolic Ca^2+^ upon treatment with haloperidol (ΔF/F_0_ (%) ± SEM = 9.4 ± 4.1) compared to treatment with the D2 agonist, quinpirole (50 μM), (ΔF/F_0_ (%) ± SEM = −9.1 ± 3.9) (*p* = 0.0091, n = 14, paired *t* test). Thus, AMPH can promote a rise in cytosolic Ca^2+^ in DArgic neurons in a manner dependent on D2 autoreceptors’ activity.

### An NMDA-elicited rise in intracellular Ca^2+^ does not promote AMPH-induced efflux

To investigate if an increase of intracellular Ca^2+^ could increase the DA efflux caused by AMPH, the neurons were incubated with 20 μM NMDA and 10 μM glycine before and during the application of 10 μM AMPH (Fig. 5*B*). As shown above (Fig. 1*B*), activation of NMDA receptors caused a conspicuous increase in intracellular Ca^2+^. However, comparison of the fluorescent change in DA signal induced by AMPH alone (ΔF/F_0_ (%) ± SEM = 10 ± 1.6) with the signal caused by AMPH when the neurons have been incubated with 20 μM NMDA and 10 μM glycine (ΔF/F_0_ (%) ± SEM = 11 ± 2.0) revealed no difference (*p* = 0.19, n = 4, paired *t* test).

### Blockade of voltage-dependent Ca^2+^-channels or chelation of cytosolic Ca^2+^ do not affect AMPH-induced efflux

Although the results presented so far have not provided evidence for a dependency of AMPH-induced DA efflux on intracellular Ca^2+^ levels, it still cannot be excluded that jRGECO1a is unable to detect Ca^2+^ fluctuations localized to subdomains of the neurons. Therefore, we tested the effect of sequestering putative increases in intracellular Ca^2+^ by using the membrane-permeable Ca^2+^ chelator 1,2-bis(2-aminophenoxy)ethane-N,N,N′,N′-tetraacetic acid tetrakis(acetoxymethyl ester) (BAPTA-AM) (Fig. 5*C*). Intriguingly, we saw no difference in the AMPH-induced DA efflux in response to 10 μM AMPH before (ΔF/F_0_ (%) ± SEM = 18 ± 1.6) and following 15 min preincubation of 50 μM BAPTA-AM (ΔF/F_0_ (%) ± SEM = 17 ± 1.8) (*p* = 0.7021, n = 3, paired *t* test). The lack of effect was not because BAPTA-AM did not chelate cytosolic Ca^2+^. As shown in Fig. S4, BAPTA-AM strongly chelated Ca^2+^ as evidenced by a marked decrease in jRGECO1a fluorescence upon application of BAPTA-AM.

We also tested the effect of blocking either L- or N-type voltage-gated calcium channels (VGCC) to exclude discrete effects of localized channel openings. No effect on DA efflux was seen upon preincubating for 5 min with L-type channel blocker nifedipine (*p* = 0.12, n = 3, paired *t* test) or the N-type blocker ω-conotoxin (*p* = 0.42, n = 3, paired *t* test) (Fig. 5, *D* and *E*). To ensure that ω-conotoxin was capable of blocking Ca^2+^ entry, we assessed its effect on the Ca^2+^ entry seen upon KCl-induced depolarization. As shown in Fig. S5, ω-conotoxin almost eliminated the rise in cytosolic Ca^2+^ induced by KCl.

### Activation of PKC or CaMKIIα is not required for AMPH-induced efflux

**Figure 6:**
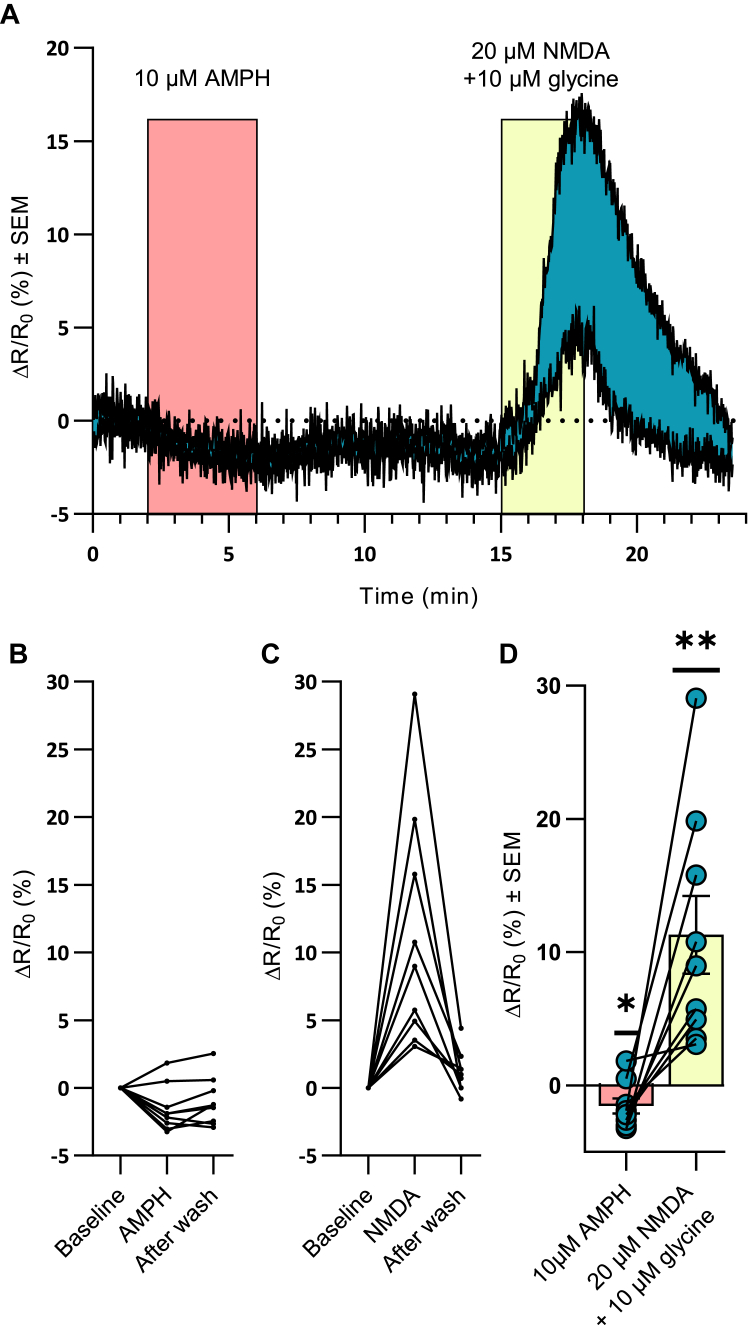
**Expression of a FRET-based reporter of CaMKIIα activation (Camuiα-CR) in DArgic neurons reveals that AMPH does not affect CaMKIIα activation**. In contrast, stimulation of NMDA receptors caused activation of CaMKIIα. *A*, representative trace of the ratiometric change (ΔR/R_0_) for the reporter of CaMKIIα activation shows no effect of 10 μM AMPH while 20 μM NMDA + 10 μM glycine increases CaMKIIα activation. *B*, ΔR/R_0_ values from individual neurons expressing Camuiα-CR before, during and after application of 10 μM AMPH. The values are from three independent experiments and are expressed as % of baseline. *C*, ΔR/R_0_ values from individual neurons expressing Camuiα-CR before, during, and after application of 20 μM NMDA + 10 μM glycine. The values are from three independent experiments and are expressed as % of baseline. *D*, the bar graph shows the mean ΔR/R_0_ ± SEM for the CaMKIIα activation reporter from individual neurons recorded during three independent experiments. CaMKIIα activation following 10 μM AMPH was not different from baseline, whereas stimulation of NMDA receptors caused an increase. AMPH, amphetamine; CaMK, calmodulin-dependent protein kinase; NMDA, N-methyl-D-aspartate.

Both PKC (16, 19, 20, 22, 23, 24) and CaMKIIα (25, 26, 45) have been implicated in AMPH-mediated DA efflux, and the activation of both kinases involves Ca^+2^. Since we did not see an effect of modulating intracellular Ca^2+^ levels in the primary midbrain cultures, we decided to investigate the putative role of the two kinases in our setup. We observed no change in the DA signal following incubation with the PKC inhibitor Go 6976 (ΔF/F_0_ (%) ± SEM = 10 ± 2.2) compared to AMPH alone (ΔF/F_0_ (%) ± SEM = 7.9 ± 1.5) (*p* = 0.20, n = 4, paired *t* test) (Fig. 5*E*). For CaMKIIα, we first investigated whether AMPH could activate the kinase by expressing the FRET-based reporter of CaMKIIα activation Camuiα-CR (46) in the DArgic neurons (Fig. 6). However, application of 10 μM AMPH to the neurons caused a small decrease to the ratiometric signal of the CaMKIIα reporter (ΔR/R_0_ (%) ± SEM = −1.6 ± 0.56, *p* = 0.024, n = 9, one-sample *t* test), indicating that AMPH did not cause activation of the kinase (Fig. 6, *B* and *D*). On the other hand, stimulation of NMDA receptors induced an increase in the ratiometric signal of the reporter for CaMKIIα activation (ΔR/R_0_ (%) ± SEM = 11 ±2.9, *p* = 0.0047, n = 9, one-sample *t* test) (Fig. 6, *C* and *D*). Additionally, we found no change in DA efflux from preincubating the neurons with the CaMKIIα inhibitor KN-93 (10 µM) before coapplication with AMPH (ΔF/F_0_ (%) ± SEM = 11 ± 3.3 with and 9.7 ± 2.2 without KN93, *p* = 0.54, n = 9, paired *t* test) (Fig. S6*A*). However, the inactive KN-93 analog, KN-92, caused an unexpected inhibition of AMPH-induced DA efflux (ΔF/F_0_ (%) ± SEM, 2.7 ± 0.58 with and 7.0 ± 0.47 without KN92, *p* = 0.03, n = 3, paired *t* test) (Fig. S6*B*), questioning the reliability of using KN93 in the assay to assess the role of CaMKIIα. To further investigate the role of CaMKIIα in AMPH-induced DA efflux we tried to use the CaMKIIα inhibitor autocamtide-2 related inhibitory peptide II. Unfortunately, we were unable to perform analysis of this experiment because we observed an unexplainable effect of this compound on baseline fluorescence from the sniffer cells.

## Discussion

Here, we use genetically encoded fluorescent sensors to establish a new method that enables parallel measurements of changes in extracellular DA and activation of intracellular signaling cascades in cultured DA neurons. The measurements become possible by combining the use of “sniffer” cells stably expressing the fluorescent DA sensor GRAB_DA1H_ with the exploitation of a double-virus approach based on the Cre-lox system to ensure specific expression of another fluorescent sensor, for example, the Ca^2+^ sensor jRGECO1a, in DA midbrain neurons themselves. By seeding the sniffer cells on top of the midbrain neuronal cultures, we can reliably measure the fluorescence from both sensors with little spatial and spectral overlap by live imaging. The method allowed us to investigate essential details relating to Ca^2+^-dependent mechanisms underlying AMPH-induced efflux in cultured DA neurons. Indeed, it could be interesting as well to perform similar experiments using acute striatal slices; however, the somatodendritic compartment will inevitably be cut off during preparation of the slice and, thus, it will not be possible to carry out the experiments on intact functional neurons as we do in this study.

Our approach revealed a substantial increase in fluorescence from the sniffer cells following the application of 10 μM AMPH, signifying an increase in extracellular DA. In support of a mechanism independent of exocytotic release, the AMPH-mediated DA signal was replicated with an even better signal-to-noise ratio when preventing neuronal firing using the Na^+^-channel blocker TTX. Under this condition, the DA efflux was dose-dependent with increasing responses to 1 μM, 10 μM, and 30 μM. Because the *K*_i_ of AMPH for rat DAT has been measured to be in the range of ∼30 nM (47), the effect of AMPH was unlikely the result of simple inhibition of DA uptake by DAT as this expectably would be saturated already at ∼1 μM AMPH. The efflux, however, was most likely DAT-dependent as it was blocked by cocaine. Of note, cocaine itself caused an increase of extracellular DA in the presence of TTX. A likely explanation is that cocaine, by blocking DAT, prevents the reuptake of DA that is released despite the presence of TTX. Indeed, some DA release may occur independently of somatic action potential firing (48). Leakage of DA by diffusion or through a different transporter/channel such as the organic cation transporter 3 (49, 50) can also not be excluded. Importantly, the response to AMPH was much larger than the response to cocaine, adding further support to the unique mechanism of action for AMPH. AMPH might also promote exocytotic vesicular release in addition to DAT mediated reverse transport (51, 52, 53), but we find it unlikely that this contributes to our current findings. Our experiments were done in the presence of TTX, and it is hard to envision that under this condition AMPH would be able to drive exocytic vesicular release, especially when there is no effect on cytosolic Ca^2+^ and no effect of VGCC blockade. Of further interest, AMPH is known to affect DAT surface expression (54); however, this is also unlikely to affect our results. The time scale of AMPH-indued DAT internalization is rather slow reaching a maximum of 40 to 50 % after 30 min (55). Additionally, after a washout period of ∼10 min, we can obtain an AMPH-response of a similar magnitude, suggesting that this period is sufficient to recover DAT surface levels (*e.g*., Fig. 5).

AMPH is a substrate for the VMAT2 (8), and in congruence with this, our data support that the AMPH-induced efflux of DA from the DAergic neurons is dependent on this transporter. The data suggest that the measured DA efflux involves the translocation of DA from the vesicle lumen to the cytosol as acute inhibition of VMAT2 hindered the efflux of DA. The data are consistent with observations in *Drosophila* supporting a crucial role of transport of AMPH by VMAT2. Conceivably, AMPH reduces the vesicle pH-gradient because of net proton antiport by VMAT out of the vesicle, which is coupled to inward transport of AMPH (11).

Several previous studies have implicated an increase in intracellular Ca^2+^ in AMPH-induced efflux *via* DAT (15, 16, 27). Nevertheless, our data provide strong evidence that AMPH-induced efflux, mediated by the action of VMAT2 and DAT together, can take place independently of changes in intracellular Ca^2+^. Additionally, the response was not affected by chelating cytosolic Ca^2+^ with BAPTA-AM or blocking L- or N-type voltage-gated Ca^2+^ channels known to be the predominant voltage-gated Ca^2+^ channels in DA neurons (56). Even boosting cytosolic Ca^2+^ by simultaneously stimulating NMDA receptors did not affect the response. Nonetheless, when pre-incubating with the D2 receptor antagonist, haloperidol, AMPH caused an increase in intracellular Ca^2+^. As we predict from our cocaine data, DA is likely spontaneously released from the dense cultures of DA neurons. This DA might stimulate the D2 autoreceptors, which in turn hyperpolarizes the neuron by promoting the opening of GIRKs and thereby inhibiting the activation of voltage-gated Ca^2+^ channels (43, 44). Blocking the D2 receptors would then release this hyperpolarizing effect and uncover an impact of AMPH on intracellular Ca^2+^. Indeed, it has been shown in HEK293 cells co-expressing DAT and VGCC that the ionic conductance from transporting AMPH is sufficient to activate L-type VGCC (42).

Our data did not provide any direct support for the requirement for activation of PKC and CaMKIIα in AMPH-mediated DA efflux in our experimental setup. Ca^2+^ can activate both kinases and both have previously been suggested to play a role in the efflux (16, 20, 21, 22, 25, 27, 57). However, by using the fluorescent sensor for CaMKIIα activation (Camuiα-CR), we found no change upon AMPH stimulation, and we observed no effect of the CaMKIIα inhibitor KN93. Unfortunately, the finding was not conclusive as we observed an unexpected inhibitory effect of the inactive KN-93 analog, KN-92. We were also not able to use the CaMKIIα inhibitor autocamtide-2 related inhibitory peptide II due to an unexplainable effect of the compound on baseline fluorescence from the sniffer cells. Importantly, we have previously shown that KN-93 can inhibit AMPH-induced DA efflux in DArgic neurons, but this was seen when the neurons were clamped to either +80 or +100 mV. Moreover, we showed that the application of activated CaMKIIα to DAergic neurons caused DA efflux on its own when the neuron was voltage-clamped to > 20 mV (25). Because the resting potential of our neurons studied conceivably is negative in the presence of TTX, and with the D2 receptor most likely being activated, it is tempting to propose that CaMKIIα, and consequently also Ca^2+^, might have a role under depolarizing conditions, such as during phasic firing. Indeed, phasic firing may commonly occur *in vivo* where previous experiments have supported the importance of CaMKIIα for the effects of AMPH. Our own previous studies showed that KN93, but not the inactive compound KN92, significantly inhibited DA efflux in a chronoamperometry experiment (25). Moreover, a microdialysis study showed reduced AMPH-induced DA efflux in CaMKIIα knock-out mice (29). Nevertheless, the current data still suggest that AMPH-induced DA efflux can occur without depolarization and Ca^2+^. Based on knock-down/molecular replacement experiments, it might even be speculated if CaMKIIα can have effects as a scaffold protein without having to be activated by Ca^2+^/calmodulin (58).

Several compounds’ ability to inhibit PKC has earlier been shown to correlate with their ability to block AMPH-induced DA efflux from striatal synaptosomes (19), but most of the compounds tested would probably not be considered specific PKC inhibitors today, for example, neomycin, spermine, and amiloride. Go 6976, however, is considered a relatively specific inhibitor of PKCα and PKCβ. Here, we did not see any effect of Go 6976 on AMPH-induced DA efflux. Perhaps, PKC, like CaMKIIα, only modulates the effect of AMPH on efflux under strong depolarizing conditions that may be achieved in, for example, slices due to the ionic conductance associated with AMPH transport. Note that in slices, the measurements are done on DA terminals that have been separated from the somatic compartment and not on intact neurons as in this study.

Summarized, we employ a live imaging approach enabling parallel monitoring of DA efflux and activation of intracellular signaling pathways in cultured DArgic neurons. Our data reveal in the neurons profound AMPH-induced DA efflux that strikingly neither requires Ca^2+^ signaling nor activation of the kinases PKC and CaMKIIα. However, the efflux depends on both the activity of VMAT2 and DAT, substantiating the unequivocal concerted importance of both transporters for the pharmacological action of AMPH on DArgic neurons.

## Experimental procedures

### Pharmacological agents

The pharmacological agents including concentrations used are listed in Table S1.

### Culturing of midbrain dopaminergic neurons

The generation of cultures were approved by the Animal Experimentation Inspectorate, Denmark (Permission 2017–15–0201–01177). All efforts were made to minimize animal suffering and to reduce the number of animals used. Wistar rat pups (Charles River) at postnatal day 1 to 2 were used as described (36) for generation of cultures of a monolayer of cortical astrocytes with midbrain dopaminergic neurons atop. The cultures were plated on 25 mm poly-D-lysine coated coverslips in 6-well plates using a serum-free medium (Neurobasal A (Gibco) with 1% GlutaMAX (Gibco), 2% B-27 plus (Gibco), 200 μM ascorbic acid, 500 μM kynurenic acid and 0.1% Pen-Strep solution (Sigma-Aldrich) and half the medium was changed twice a week. The astrocytes were grown until 70 to 80% confluent following the addition of 6.7 μg/ml 5-fluoro-2′-deoxyuridine to inhibit further cell division. Rat glial cell-derived neurotrophic factor was included two hours after plating midbrain neurons for a final concentration of 10 ng/ml. The cultures were used for imaging after 14 to 21 days *in vitro*.

### Culturing and maintenance of GRAB_DA1H_ expressing sniffer cells

GRAB_DA1H_ expressing sniffer cells were generated using Flp-In T-REx 293 cells and maintained as described (36). The cells were grown in Dulbecco's modified Eagle's medium supplemented with 10% fetal bovine serum, 200 μg/ml hygromycin B (Sigma-Aldrich) and 15 μg/ml blasticidin. One to two days before imaging, the GRAB_DA1H_ sniffer cells were seeded out on top of primary DAergic neuron cultures at a density of 2 × 10^5^ cells/well together with the application of tetracycline for a final concentration of 1 μg/ml to induce sensor expression.

### Plasmid constructs and AAV particle production and transduction

AAV1 particles from Cre-dependent constructs containing the Ca^2+^ sensor jRGECO1a (pAAV.Syn.Flex.NES-jRGECO1a.WPRE.SV40) or the axonally targeted Ca^2+^ sensor axon-GCaMP6s (pAAV-hSynapsin1-FLEx-axon-GCaMP6s) were obtained from Addgene (cat. #100853 and #11201, respectively). The construct containing Cre recombinase driven by a truncated tyrosine hydroxylase promotor, pAAV-pTH-iCre-WPREpA, was cloned as described (56). The pAAV-hSyn-DIO-Camui_CR-CW3SL construct was generated by restriction site cloning of the ORF from pcDNA3-Camui-CR (46) (Addgene, cat. #40256) into a Cre-dependent AAV backbone plasmid containing CW3SL, a compressed 3′ untranslated region, from the construct pAAV-CW3SL-EGFP (59) (Addgene, cat. #61463). Production of AAV particles in-house was done as described (56). AAV particles were applied to primary midbrain cultures after 3 days *in vitro* at a ∼2 × 109 vg/ml concentration.

### Live imaging and data calculation

Imaging was carried out using an ECLIPSE Ti-E epifluorescence microscope (NIKON) with a 488 and 561 nm laser (Coherent) and an S Plan Fluor ELWD 20X/0.45 ADM microscope objective (NIKON). Emitted light was collected with an iXon3 897 Electron Multiplying CCD camera (Andor). The neurons were mounted on a RC-21BDW imaging chamber on the microscope (Warner Instruments) with continuous perfusion with aCSF (in mM: NaCl, 120; KCl, 5; glucose, 30; MgCl_2_, 2; CaCl_2_, 2; Hepes, 25; pH 7.40) at 37 °C. GRAB_DA1H_ and axon-GCaMP6s signal were imaged with the 488 nm laser using a 505 to 545 nm bandpass filter and jRGECO1a was imaged with the 561 nm laser using a 578 to 625 nm bandpass filter. Camui-CR was imaged with a 488 nm laser and quad-band laser filter set filtering emitted light at 425 to 475, 505 to 545, 578 to 625, and 664 to 787 nm followed by a 550 nm long-pass beam-splitter leading onto two different cameras for simultaneous detection of the FRET donor/acceptor pair. All imaging data were analyzed by the ImageJ software distribution Fiji (60) (https://imagej.net/software/fiji/). Quantification of the DA signal from the GRAB_DA1H_ expressing sniffer was done by drawing regions of interest (ROIs) around clusters of cells, typically ∼5 clusters of cells in a single recording and assessing fluorescent intensity as a function of time with the ImageJ plugin Time Series Analyzer. The average signal from the different ROIs was considered a single N. Experiments were usually repeated three times (giving rise to an N of 3) on at least two independently prepared neuronal cultures. Quantification of the Ca^2+^ and Camui data was done by drawing ROIs around single somas or individual neurite fractions (typically 3–4 per experiment) and assessing fluorescent intensity as a function of time with the ImageJ plugin Time Series Analyzer. Each drug response was calculated as the area under the curve during the last minute of drug treatment normalized to baseline defined as the area under the curve 1 minute prior to the given drug treatment.

### Statistics

All graphs and statistics were done in GraphPad Prism 9 (https://www.graphpad.com). All data sets were tested for outlier using Grubb’s test. A one-sample *t* test was used for assessing if a change in fluorescence was significantly different from baseline fluorescence. A paired *t* test was used when comparing the DA signal from AMPH with or without drugs of interest. A one-way ANOVA was used for testing dose-response relationship between AMPH, and DA and Ca^2+^ signal, respectively. For post hoc analyses, we used the Holm–Šídák test. For all analyses, the significance level was set at 0.05, and all data are presented as the mean ± SEM.

## Data availability

All data relating to this manuscript is contained in the manuscript.

## Supporting information

This article contains supporting information.

## Conflict of interest

The authors declare that they have no conflicts of interest with the contents of this article.
