# Supplementary material for: Amphetamine-induced reverse transport of dopamine does not require cytosolic Ca2+
Source: J Biol Chem. 2023 Jul 18;299(8):105063. doi: 10.1016/j.jbc.2023.105063 (PMC10448275; doi:10.1016/j.jbc.2023.105063)
Supplement: Supporting information [file mmc1.pdf]

SUPPORTING INFORMATION

Supplementary Table S1

Compound	Concentrations used	Manufacturer	Cat. #
Autocamtide-2 Related Inhibitory Peptide II	10 µM	Sigma-Aldrich	189485
D-Amphetamine	1-30 µM	Sigma-Aldrich	A5880
BAPTA-AM	50 µM	Tocris	2787
Cocaine	100 µM	Copenhagen University Hospital Pharmacy	721927
Dopamine	10 µM	Sigma-Aldrich	H8502
Glycine	10 µM	Sigma-Aldrich	G8898
Go 6976	1 µM	Tocris	2253
Haloperidol	20 nM	Sigma-Aldrich	H1512
KN-92	10 µM	Tocris	4130
KN-93	10 µM	Tocris	1278
Nifedipine	30 µM	Sigma-Aldrich	N7634
N-methyl-D-aspartate	20 µM	Sigma-Aldrich	M3262
Potassium chloride	20 mM	Sigma-Aldrich	P3911
Quinpirole	50 µM	Sigma-Aldrich	Q102
Reserpine	3 µM	Sigma-Aldrich	R-0875
Ro-31 8220	1 µM	Tocris	2002
Ro-4-1284	3 µM	Sigma-Aldrich	R9157
Tetrodotoxin	1 µM	Tocris	1078
ω-conotoxin	100 nM	Tocris	1085

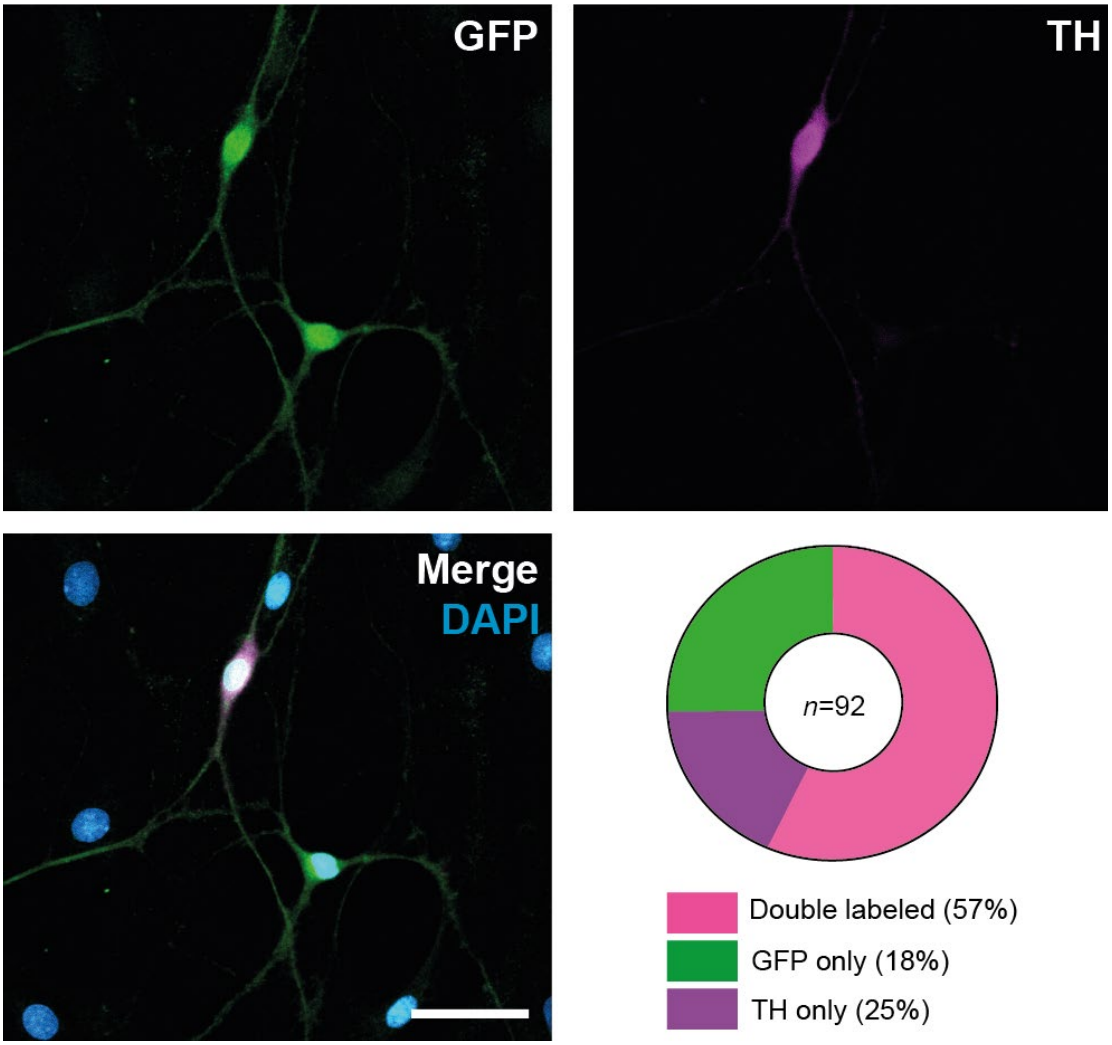

Fig. S1. AAV induced TH promoter driven Cre expression shows specificity for DA neurons in primary midbrain cultures. DA neuronal cultures were transduced with AAVs encoding TH-cre and GFP (DIO-GCaMP6s). Fourteen days post transduction, cultures were fixed and immunostained for GFP (green) and TH (purple). Quantification of 5 cultures (4 random ROIs from each culture) revealed 92 neurons, with 57% dual positive for TH and GFP (52 cells, magenta), 18% GFP only (17 cells, Green) and 25% TH only (23 cells, purple). This means that ~3 out of 4 cells (52/69) or 75 % would report correctly as the TH only positive cells would not be visible in the live imaging experiments. Scalebar = 50um.

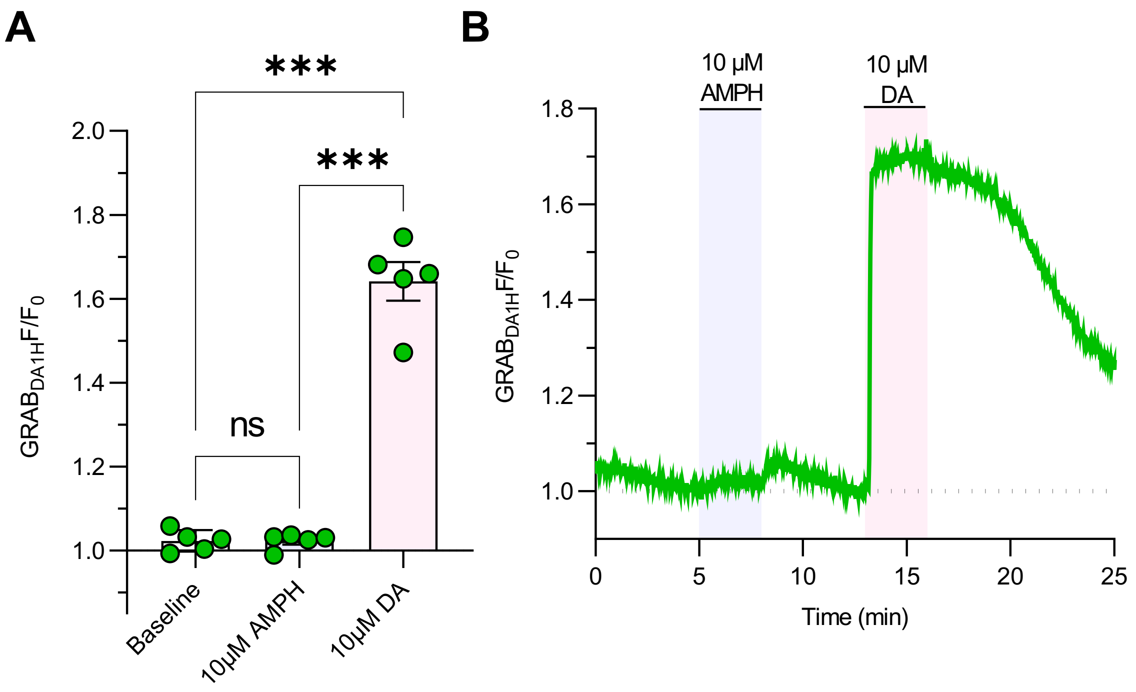

Fig. S2. AMPH does not have an effect on $GRAB_{DA1H}$ -expressing "sniffer" cells. a. The bar graph shows the change in DA-dependent fluorescent signal of $GRAB_{DA1H}$ sniffer cells co-cultured with midbrain DA neurons from baseline upon adding 10 µM AMPH and 10 µM Dopamine as a control. Data is presented as mean responses of the mean of F/F_0 ± SEM, n=5. ns, $P > 0.05$; *, $P \leq 0.05$, **$p \leq 0.01$; one-way ANOVA, Holm-Šídák post hoc test. **b.** Representative trace of $GRAB_{DA1H}$ signal upon the different drug treatments, as indicated with colored backgrounds.

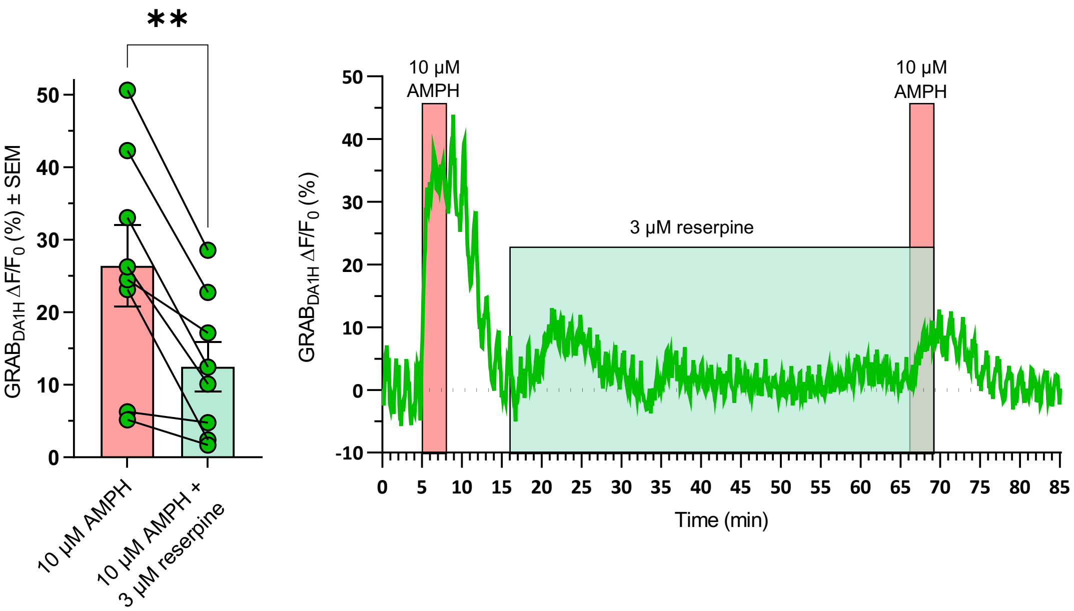

Fig. S3 Pre-incubation with the VMAT2 inhibitor reserpine blocks AMPH-induced DA efflux. (Left) The bar graph shows the change in the DA-dependent fluorescent signal of GRAB$_{DA1H}$ sniffer cells co-cultured with midbrain DA neurons following application of 10 µM AMPH with and without pre-incubation with 3 µM reserpine. Values are average changes in DA signal from baseline during the last minute of AMPH treatment expressed as $\Delta F/F_0$ in % ± SEM, n=3. (Right) Representative trace of the change in DA signal from neurons treated with AMPH alone or after pre-incubation with reserpine, **$P<0.01$; paired t-test.

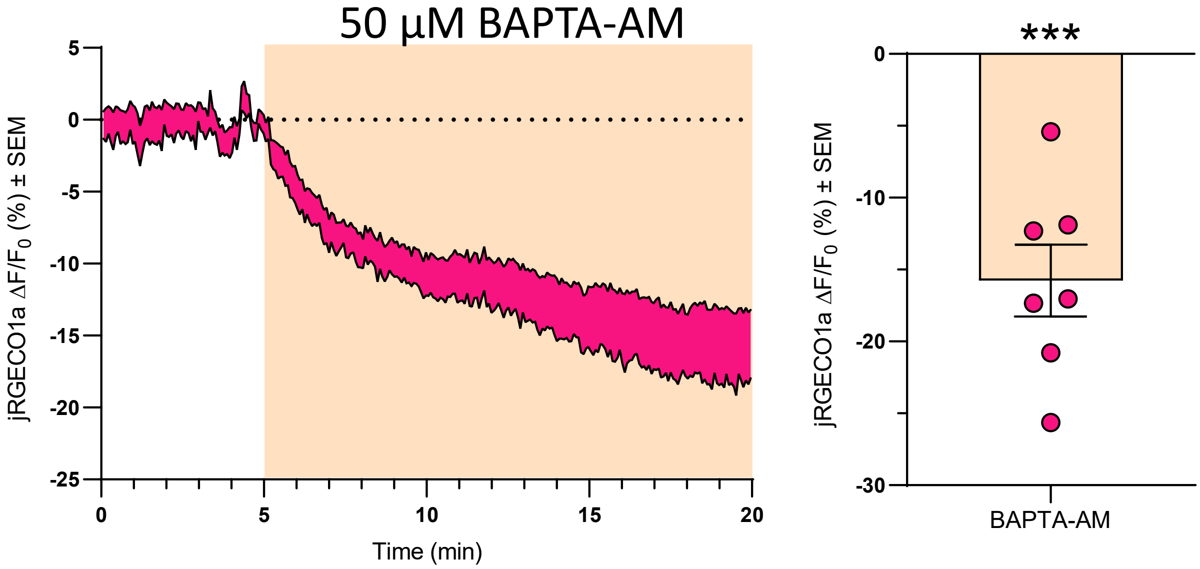

Fig. S4. Treatment with BAPTA-AM in the presence of TTX chelates intracellular Ca^{2+}. (Left) Change in Ca^{2+} signal from jRGECO1a expressed in midbrain DA neurons upon application of 50 µM BAPTA AM as indicated. Data shown are an average trace of 7 neurons from three independent experiments ± SEM. The bar graph (right) represents the average change in fluorescence from baseline measured from the last minute during a 15 minutes BAPTA-AM treatment, *** $P \leq 0.001$, one-sample t-test.

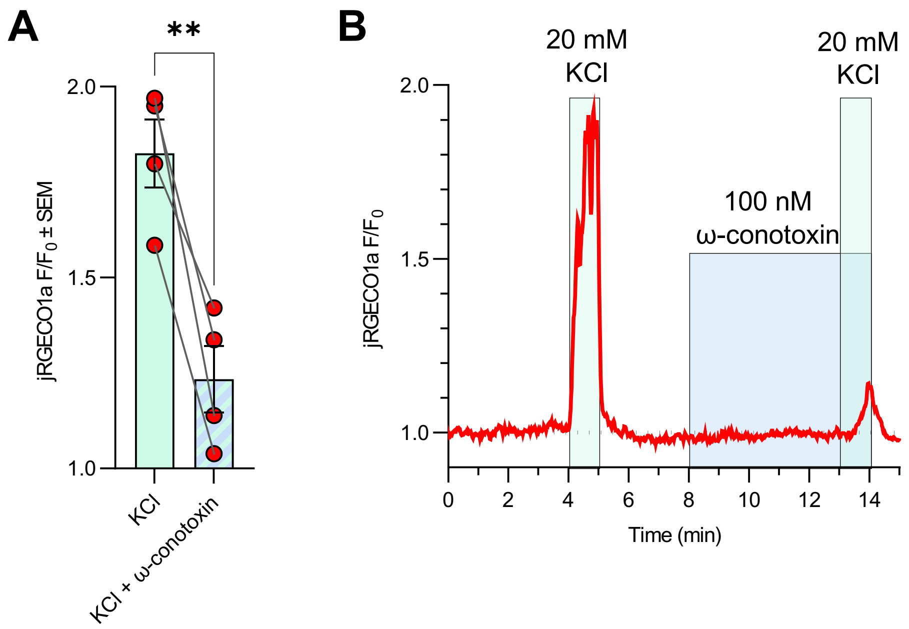

Fig S5. Incubation with 100nM ω-conotoxin reduces the rise of intracellular Ca^{2+} upon KCl depolarization. a. Bar graph of the change in the Ca^{2+}-dependent fluorescent signal from jRGECO1a expressed in midbrain DA neurons when applying 20mM KCl in the absence or presence of the N-type voltage-gated calcium channel inhibitor, ω-conotoxin, compared to baseline. Data is plotted as max response per condition as $F/F_0 \pm$ SEM, n=4. **b.** Representative trace of the effect of ω-conotoxin on intracellular calcium, *, $p < 0.05$; **, $p \leq 0.01$, paired t-test.

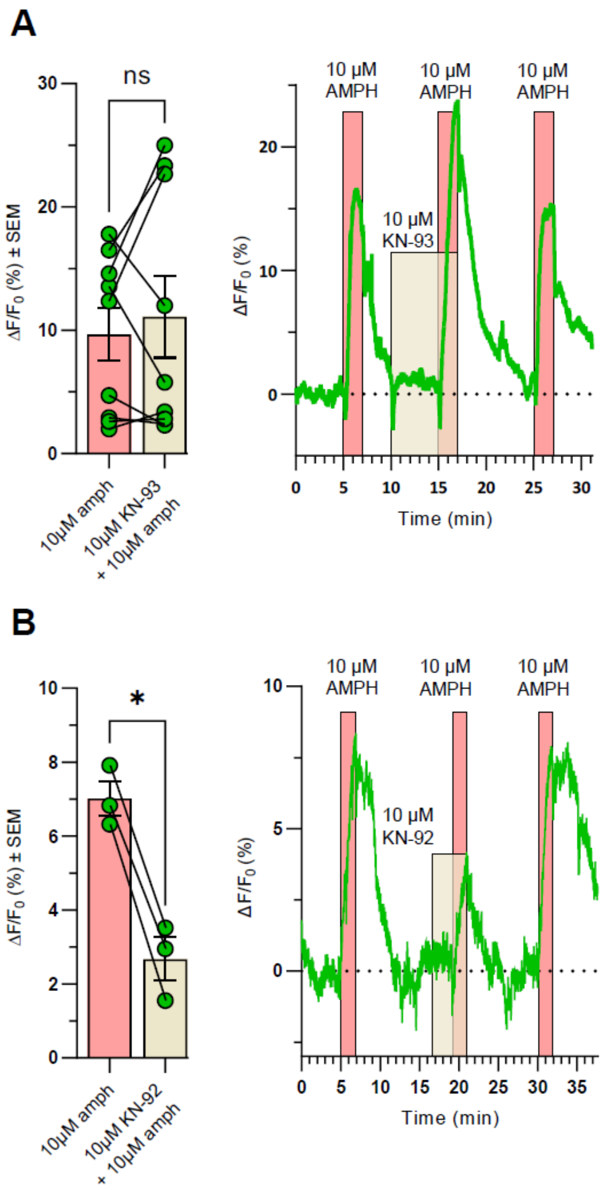

Fig S6. The inactive KN-93 analog, KN-92, inhibits AMPH-induced DA efflux indicating potential unappreciated off-target effects of the CAMKIIα inhibitor. a. Pre-incubation with 10 µM CaMKIIα inhibitor KN-93 did not affect AMPH-induced DA efflux. (Left) The bar graph shows the change in the DA-dependent fluorescent signal of $GRAB_{DA1H}$ following application of AMPH with and without pre-incubation with KN-93. Data are mean of $\Delta F/F_0$ in % ± SEM, n=9. (Right) Representative trace **b.** KN-92 inhibits AMPH-mediated DA efflux. (Left) The bar graph shows the change in the DA-dependent fluorescent signal of $GRAB_{DA1H}$ following application of

AMPH with and without pre-incubation with KN-92. Data are mean of $\Delta F/F_0$ in % ± SEM, n=3. (Right) Representative trace. ns $P > 0.05$; * $P < 0.05$, paired t-test.
